# Effects of Nutritional Supplements on Vascular Function: A Narrative Review

**DOI:** 10.3390/jcm15145693

**Published:** 2026-07-20

**Authors:** Eleni Katsanaki, Georgios Georgiopoulos, John Thymis, George Pavlidis, Konstantinos Katogiannis, Panagiota Efstathia Nikolaou, Dimitrios Vlastos, John Parissis, Vaia Lambadiari, Ignatios Ikonomidis

**Affiliations:** 1 2nd Department of Cardiology, Attikon University Hospital, Medical School, National and Kapodistrian University of Athens, 12462 Athens, Greece; helenkatsanaki@gmail.com (E.K.); georgiopoulosgeorgios@gmail.com (G.G.); johnythg@gmail.com (J.T.); geo_pavlidis@yahoo.gr (G.P.); kenndj89@gmail.com (K.K.); dimitrisbvr@hotmail.com (D.V.); jparissis@yahoo.com (J.P.); 2 Laboratory of Pharmacology, School of Pharmacy, National and Kapodistrian University of Athens, 15771 Athens, Greece; nayanikolaou@hotmail.com; 3 Research Unit and Diabetes Center, 2nd Department of Internal Medicine, Attikon University Hospital, Medical School, National and Kapodistrian University of Athens, 12462 Athens, Greece; vlambadiari@gmail.com

**Keywords:** endothelial function, vascular function, nutritional supplements, nutraceuticals, nitric oxide, oxidative stress, arterial stiffness, flow-mediated dilation, coenzyme Q10, L-citrulline

## Abstract

Vascular aging is a key determinant of cardiovascular risk and is characterized by endothelial dysfunction, oxidative stress, inflammation, arterial stiffness, and alterations of the endothelial glycocalyx. In recent years, increasing scientific interest has focused on whether selected nutritional supplements can modulate these mechanisms. The objective of this narrative review is to summarize and critically evaluate the current evidence regarding the effects of selected nutritional supplements on endothelial function and vascular health, with emphasis on their proposed mechanisms of action, available preclinical and clinical evidence, and current limitations. This review examines nutritional supplements targeting nitric oxide biology (L-arginine/L-citrulline and citrulline derived from watermelon), polyphenol-rich foods and extracts (cocoa flavan-3-ols and grape polyphenols), mitochondrial redox balance (coenzyme Q10 and the mitochondria-targeted antioxidant MitoQ), endothelial glycocalyx-supporting formulations, olive-derived bioactive compounds, curcumin, and S-allyl cysteine. Across studies, improvements have been reported primarily in surrogate vascular endpoints, including flow-mediated dilation, pulse wave velocity, and blood pressure, although effect sizes vary and substantial heterogeneity exists among studies. Overall, current evidence suggests that several nutritional supplements may improve surrogate markers of vascular function; however, evidence demonstrating reductions in major cardiovascular events or cardiovascular mortality remains limited. Future research should prioritize adequately powered randomized controlled trials with longer follow-up, standardized supplement formulations, and clinically meaningful cardiovascular outcomes to more clearly define the role of nutritional supplements in cardiovascular prevention.

## 1. Introduction

Cardiovascular diseases (CVDs) remain the leading cause of morbidity and mortality worldwide. Consequently, considerable research efforts have focused on identifying the mechanisms and modifiable risk factors involved in their development. Although numerous cardiovascular risk factors have been identified, hypertension, diabetes mellitus, dyslipidemia, obesity, and smoking remain among the most important modifiable contributors to cardiovascular disease. Exposure to these cardiovascular risk factors promotes inflammation, oxidative stress, and vascular immune dysfunction, which collectively contribute to endothelial injury and atherosclerosis. Approximately 80% of coronary heart disease is considered preventable through adherence to a healthy lifestyle, including a balanced diet, regular physical activity, weight management, moderation of alcohol consumption, and smoking cessation. Nevertheless, contemporary dietary patterns and lifestyle behaviors continue to contribute to the increasing prevalence of cardiovascular risk factors. Accordingly, increasing attention has been directed toward nutritional supplements with the potential to improve endothelial function and vascular health as adjuncts to established lifestyle and pharmacological interventions [1]. In addition to lifestyle interventions, assessment of biomarkers reflecting endothelial dysfunction, oxidative stress, and inflammation may help identify individuals at increased cardiovascular risk and guide preventive strategies [2,3]. Among these strategies, nutritional supplementation has emerged as a potential adjunctive approach for improving endothelial function. Endothelial dysfunction is the initial step in the pathogenesis of atherosclerosis, which leads to cardiovascular and cerebrovascular events. Endothelial dysfunction is particularly pronounced in patients with hypertension, diabetes mellitus, coronary artery disease, and peripheral artery disease [4]. Thus, it is important to select appropriate interventions to prevent endothelial dysfunction in those patients. Nutritional supplementation represents one potential adjunctive intervention for improving endothelial function. Several studies have demonstrated that supplementation with L-arginine (a substrate of nitric oxide [NO]), flavan-3-ols, olive oil, fish oil, mitochondria-targeted antioxidants, other antioxidant polyphenols, and formulations containing glycosaminoglycans and fucoidan may improve endothelium-dependent vasodilation in both healthy individuals and patients with cardiovascular disease.

## 2. Literature Search Methodology

This narrative review was conducted using a structured but non-systematic literature search strategy. Electronic databases including PubMed/MEDLINE, Scopus, and Google Scholar were searched for relevant articles published from January 2000 to June 2025. The final literature search was performed on 30 June 2025.

The search strategy included combinations of the following keywords: endothelial function, vascular function, vascular aging, endothelial dysfunction, dietary supplements, nutraceuticals, L-arginine, L-citrulline, watermelon, flavan-3-ols, cocoa flavanols, coenzyme Q10, MitoQ, grape polyphenols, Taurisolo, glycocalyx, Endocalyx-Pro, olive polyphenols, oleuropein, hydroxytyrosol, black garlic, and S-allyl cysteine.

Priority was given to randomized controlled trials, systematic reviews, meta-analyses, and high-quality mechanistic studies investigating the effects of dietary supplements on endothelial function, arterial stiffness, nitric oxide bioavailability, oxidative stress, inflammation, or endothelial glycocalyx integrity. Reference lists of relevant articles were also screened to identify additional eligible studies.

Studies were included if they were published in English and evaluated dietary supplements with potential vascular effects in either human participants or, where appropriate, mechanistic animal and cellular studies that contributed to understanding biological mechanisms. Studies unrelated to vascular outcomes, conference abstracts without full-text availability, editorials, and duplicate publications were excluded.

Because the objective of the present review was to provide a focused overview of nutritional supplements supported by the strongest available evidence and emerging mechanistic data, the literature search was intentionally selective rather than fully systematic. Consequently, this review should be interpreted as a narrative synthesis rather than a comprehensive systematic review.

The nutritional supplements included in this review were selected based on the availability of human clinical evidence and mechanistic data supporting potential effects on endothelial function, arterial stiffness, nitric oxide bioavailability, oxidative stress, inflammation, or endothelial glycocalyx integrity. Particular emphasis was placed on compounds supported by randomized controlled trials, meta-analyses, or emerging translational evidence. Therefore, this review was not intended to provide an exhaustive overview of all nutraceuticals with potential cardiovascular effects, but rather to present representative nutritional supplements supported by the strongest available clinical and mechanistic evidence.

## 3. The Role of L-Arginine and L-Citrulline in Endothelial Nitric Oxide Synthesis

Adequate availability of L-arginine (L-ARG) in endothelial cells is essential for the synthesis of nitric oxide (NO), a potent vasodilator and key regulator of blood pressure and organ perfusion. As the sole substrate for endothelial nitric oxide synthase (eNOS), L-ARG plays a critical role in maintaining vascular homeostasis. However, under conditions such as aging, hypertension, and obesity, increased arginase activity diverts L-ARG metabolism away from NO synthesis, leading to L-ARG deficiency and subsequent endothelial dysfunction. L-citrulline (L-CIT), a non-proteinogenic amino acid that is not metabolized by arginase, serves as an effective precursor to L-ARG and enhances NO production. Oral L-CIT supplementation has been shown to increase circulating L-ARG levels, thereby increasing NO bioavailability and supporting vascular function. Watermelon, particularly the rind, is a natural source of both L-citrulline (L-CIT) and L-arginine (L-ARG). Evidence suggests that dietary L-citrulline derived from watermelon or from supplements may improve endothelial function by restoring nitric oxide (NO) synthesis, highlighting its potential role in maintaining vascular homeostasis and preventing endothelial dysfunction [5,6,7].

### 3.1. Watermelon (L-Citrulline-Rich) Supplementation and Peripheral Vascular Function

The effects of watermelon supplementation on vascular health have been investigated in several randomized controlled trials and comprehensively summarized in a recent meta-analysis of 10 randomized controlled trials (RCTs), which evaluated surrogate markers of vascular function, primarily arterial stiffness (Table 1). The included studies were conducted in healthy adults and individuals with prehypertension or obesity, with intervention durations ranging from 16 days to 6 weeks. L-citrulline doses from watermelon ranged from 2.7 to 6 g/day, with some studies administering watermelon juice providing approximately 3.4 g/day of L-citrulline. Postprandial studies evaluated doses of 0.8–6.9 g L-citrulline, with follow-up periods ranging from 45 min to 8 h after consumption. Regarding vascular outcomes, long-term watermelon consumption significantly reduced arterial stiffness (PWV −0.9 m/s; *p* < 0.001), while other vascular function markers (AIx, MAP, PWA) did not show significant changes. Postprandial effects on blood flow were negligible. In terms of cardiometabolic markers, watermelon consumption did not affect long-term glucose levels but significantly lowered postprandial glucose (−0.6 mmol/L, *p* < 0.001). No changes were observed in cholesterol or insulin. Watermelon consumption significantly increased plasma L-arginine (+49.0 µmol/L, *p* < 0.001) and also raised L-citrulline levels, suggesting enhanced nitric oxide (NO) bioavailability. All included studies were assessed as having a low risk of bias, and according to the GRADE assessment, the certainty of the evidence ranged from moderate to high. Overall, evidence from randomized controlled trials suggests that watermelon supplementation may improve surrogate markers of vascular function, particularly arterial stiffness, while also improving postprandial glucose responses and increasing circulating L-arginine and L-citrulline concentrations. However, current evidence is based primarily on intermediate vascular outcomes rather than major cardiovascular events, and additional large-scale, long-term randomized controlled trials are required to determine whether these improvements translate into clinically meaningful cardiovascular benefits [5,7].

### 3.2. L-Citrulline Supplementation and Cardiovascular Outcomes in At-Risk Populations

**Figure 1:**
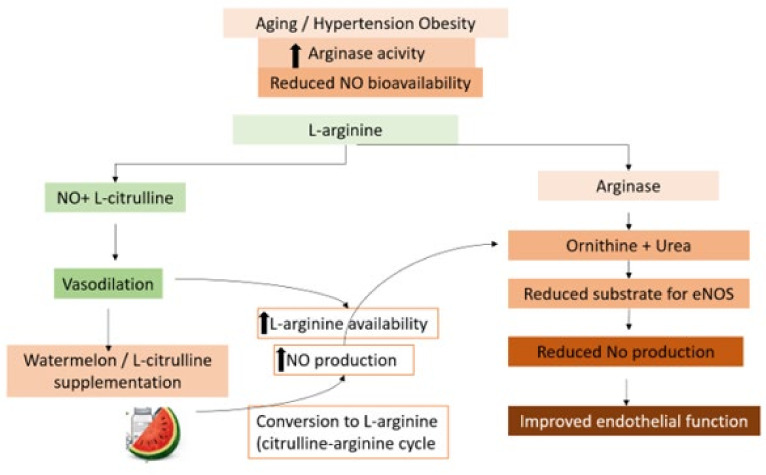
Schematic representation of the L-arginine–nitric oxide pathway and the role of L-citrulline supplementation in endothelial function. Under physiological conditions, L-arginine serves as the substrate for endothelial nitric oxide synthase (eNOS), which catalyzes the production of nitric oxide (NO) and L-citrulline. Nitric oxide is a key mediator of endothelium-dependent vasodilation and vascular homeostasis. In conditions such as aging, hypertension, and obesity, arginase activity is increased, competing with eNOS for available L-arginine by converting it into ornithine and urea, thereby reducing substrate availability for NO synthesis. This imbalance contributes to decreased NO bioavailability and endothelial dysfunction. Supplementation with L-citrulline, either directly or through natural sources such as watermelon, enhances circulating L-arginine levels via the citrulline–arginine recycling pathway, leading to increased NO production and improved endothelial function. The schematic illustration of the watermelon included in the figure was created with the assistance of ChatGPT-5 (OpenAI, https://chatgpt.com; accessed on 20 May 2025). Black arrows indicate the direction of the metabolic pathway and the relationships between components. Green boxes indicate physiological or beneficial processes, whereas orange boxes indicate pathological processes or mechanisms contributing to endothelial dysfunction. Abbreviations: eNOS, endothelial nitric oxide synthase; NO, nitric oxide.

L-citrulline (L-CIT) supplementation has been extensively investigated for its potential benefits on endothelial function, arterial stiffness, and blood pressure regulation across a range of populations. Evidence from randomized controlled trials suggests that L-citrulline supplementation may improve these surrogate markers of vascular function. For instance, in a randomized controlled trial by Maharaj et al. (2022) [8], postmenopausal hypertensive women receiving 10 g/day of L-CIT for four weeks exhibited improved endothelial function, as evidenced by increased brachial artery flow-mediated dilation (FMD), alongside reductions in resting aortic diastolic and mean arterial pressure. While the decrease in carotid–femoral pulse wave velocity (cfPWV) did not reach statistical significance, a trend toward reduced aortic stiffness was noted. Similarly, Jaime et al. reported that 6 g/day of L-CIT for 14 days in older adults significantly attenuated brachial and aortic systolic blood pressure, pulse pressure, and wave reflection during cold-induced sympathetic stimulation, suggesting favorable vascular responses during sympathetic stress. In patients with type 2 diabetes mellitus (T2DM), where vascular dysfunction is linked to reduced nitric oxide (NO) availability due to increased arginase activity, L-CIT supplementation (2 g/day for one month) was associated with a 21% reduction in arginase activity and a 38% increase in plasma NO levels [9]. These changes were further supported by in vitro findings demonstrating restoration of NO production under hyperglycemic conditions. Supporting these findings, a meta-analysis by Allerton et al. reported that L-CIT supplementation was associated with a modest but significant reduction in systolic blood pressure (~4 mmHg), with improvements in diastolic blood pressure being dose-dependent, particularly at doses ≥6 g/day. The authors proposed enhanced NO bioavailability and reduced sympathetic vasomotor tone as likely mechanisms, while emphasizing the need for larger, well-controlled trials because of the heterogeneity among existing studies. Overall, these findings suggest that L-citrulline supplementation may improve endothelial function and blood pressure, particularly in populations at increased cardiovascular risk. However, the available evidence is derived primarily from studies evaluating surrogate vascular outcomes rather than major cardiovascular events or cardiovascular mortality [8,9,10,11,12]. The main characteristics are summarized in Table 2 and Figure 1.

## 4. The Role of Flavan-3-Ols in Endothelial Function and Cardiovascular Disease Prevention

Plant-based dietary patterns have been consistently associated with a reduced risk of cardiovascular disease (CVD), highlighting the importance of adhering to healthy diets in mitigating cardiovascular risk factors [13,14]. Beyond overall dietary patterns, increasing attention has been directed toward the role of specific bioactive compounds in cardiovascular protection [15,16]. Among these compounds, (poly)phenols—particularly flavan-3-ols (flavanols)—have attracted considerable interest because of their beneficial effects on cardiovascular health [17]. Flavan-3-ols represent an important subclass of flavonoids widely present in fruits, green tea, red wine, and cocoa [18]. This subclass comprises a diverse range of monomeric, oligomeric, and polymeric compounds, including monomers such as (+)-catechin, (−)-epicatechin, (+)-gallocatechin, (−)-epigallocatechin, and their gallate derivatives. Flavan-3-ols modulate multiple cellular signaling pathways involved in endothelial function and vascular homeostasis [19,20,21].

Cardiovascular disease is frequently characterized by impaired endothelial function associated with a pro-inflammatory environment and increased cytokine production [22,23]. Such an inflammatory milieu may be triggered by oxidative stress, arising from an imbalance between the production of reactive oxygen species (ROS) and the endogenous antioxidant defense system, ultimately leading to cellular damage and death [24]. The protective effects of flavan-3-ols are largely attributed to their potent antioxidant properties, which involve both direct and indirect mechanisms [25,26]. Directly, flavan-3-ols can donate electrons to neutralize ROS and inhibit radical formation [27,28], as well as chelate metal ions that catalyze oxidative reactions [29]. Indirectly, they regulate the expression and activity of key antioxidant enzymes, including catalase (CAT), superoxide dismutase (SOD), and glutathione peroxidase (GSH), thereby enhancing cellular antioxidant capacity [30,31,32].

**Figure 2:**
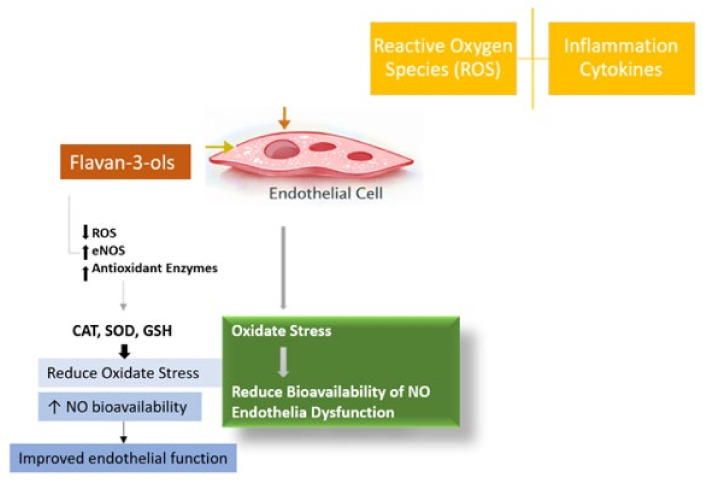
Schematic representation of the protective effects of flavan-3-ols on endothelial function under conditions of oxidative stress and inflammation. Reactive oxygen species (ROS) and inflammatory cytokines promote oxidative stress in endothelial cells, leading to reduced nitric oxide (NO) bioavailability and endothelial dysfunction. Flavan-3-ols exert vasoprotective effects by decreasing ROS levels, enhancing endothelial nitric oxide synthase (eNOS) activity, and upregulating endogenous antioxidant defense systems. In particular, flavan-3-ols increase the activity of antioxidant enzymes such as catalase (CAT), superoxide dismutase (SOD), and glutathione (GSH), thereby contributing to the reduction in oxidative stress. These mechanisms collectively improve NO bioavailability and support endothelial function. The endothelial cell illustration included in the figure was created with the assistance of ChatGPT-5 (OpenAI, https://chatgpt.com; accessed on 20 May 2025). Black arrows indicate the direction of the biological pathway and interactions. Green boxes indicate protective or beneficial effects, whereas orange boxes indicate pathological stimuli or detrimental processes. Upward (↑) and downward (↓) arrows indicate increased and decreased levels or activity, respectively. Abbreviations: ROS, reactive oxygen species; eNOS, endothelial nitric oxide synthase; NO, nitric oxide; CAT, catalase; SOD, superoxide dismutase; GSH, glutathione.

Between 2003 and 2020, 35 experimental studies, randomized controlled trials, and meta-analyses evaluated the effects of flavan-3-ol supplementation on cardiovascular health. Most studies demonstrated improvements in flow-mediated dilation (FMD), reductions in pulse wave velocity (PWV), and decreases in systolic and diastolic blood pressure, suggesting favorable effects on surrogate markers of vascular function. However, current evidence is derived predominantly from studies evaluating surrogate vascular endpoints rather than major cardiovascular events or cardiovascular mortality. A summary of the principal clinical studies is presented in Table 3 and Figure 2 [33].

## 5. The Role of Coenzyme Q10 Supplementation in Enhancing Endothelial Function and Cardiovascular Health

Coenzyme Q10 (CoQ10) supplementation has attracted considerable attention because of its potential benefits for vascular health, particularly endothelial function. CoQ10 is a lipid-soluble quinone that plays an essential role in mitochondrial electron transport and acts as a potent antioxidant, protecting cellular membranes against oxidative stress, a key contributor to cardiovascular disease (CVD), dyslipidemia, hypertension, and endothelial dysfunction [38,39,40]. A recent systematic review and meta-analysis of randomized controlled trials by Daei et al. (2024), which included 12 clinical trials involving 489 participants, evaluated the effects of CoQ10 supplementation on endothelial function and vascular health [41]. The meta-analysis demonstrated a significant improvement in flow-mediated dilation (FMD), a surrogate marker of endothelial function, particularly among individuals older than 55 years, those with a body mass index (BMI) ≥26 kg/m^2^, and participants receiving supplementation for at least 8 weeks.

The meta-analysis found no significant changes in intercellular adhesion molecule-1 (ICAM-1) or vascular cell adhesion molecule-1 (VCAM-1), consistent with previous studies reporting limited effects on these inflammatory markers. The observed improvement in FMD may be explained by the antioxidant properties of CoQ10, which may enhance nitric oxide (NO) bioavailability, reduce oxidative stress, and attenuate inflammatory signaling through inhibition of NF-κB activation and downstream pro-inflammatory cytokines. In addition, CoQ10 supports mitochondrial bioenergetics, which may further contribute to improved vascular function [38,41,42,43,44].

CoQ10 supplementation appears to be generally safe, with only minor gastrointestinal adverse effects reported, and doses of up to 1200 mg/day have been well tolerated in patients with cardiovascular disease.

Despite the strengths of the available evidence, including high-quality randomized controlled trials and sensitivity analyses, limitations such as relatively small sample sizes and heterogeneity among studies warrant cautious interpretation of the findings, particularly regarding ICAM-1 and VCAM-1. Furthermore, individual studies, such as that by Raitakari et al., did not observe significant improvements in FMD despite increased circulating CoQ10 concentrations, suggesting that treatment duration, dosage, or patient characteristics may influence the vascular response to supplementation.

**Figure 3:**
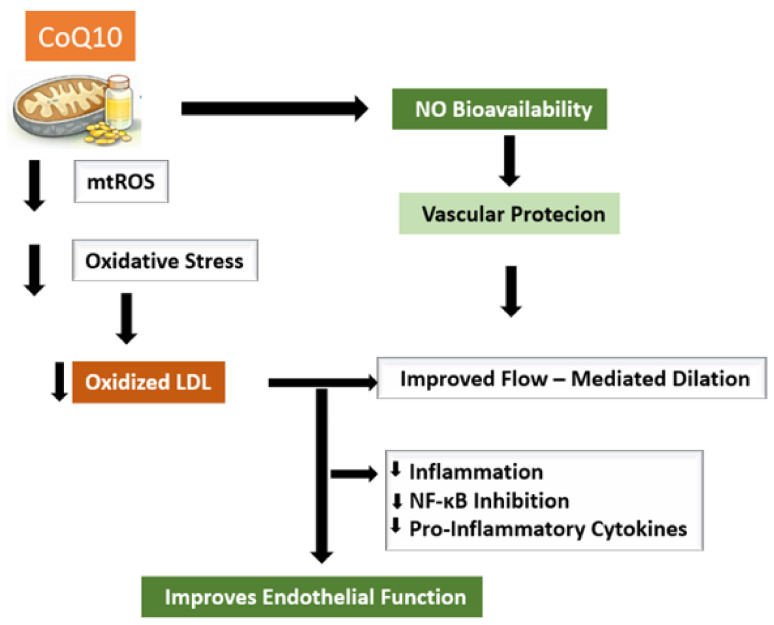
Schematic illustration of the vascular protective effects of coenzyme Q10 (CoQ10) on endothelial function. CoQ10 acts as a mitochondrial antioxidant, reducing mitochondrial reactive oxygen species (mtROS) production and attenuating oxidative stress. This leads to decreased oxidation of low-density lipoproteins (oxLDL) and increased nitric oxide (NO) bioavailability. Enhanced NO signaling improves vascular protection and flow-mediated dilation (FMD). Additionally, CoQ10 exerts anti-inflammatory effects through inhibiting nuclear factor-kappa B (NF-κB) signaling and reducing pro-inflammatory cytokine production, ultimately contributing to improved endothelial function. The schematic illustration of the CoQ10 compound included in the figure was created with the assistance of. ChatGPT-5 (OpenAI, https://chatgpt.com; accessed on 20 May 2025). Black arrows indicate the direction of the proposed biological effects. Upward (↑) and downward (↓) arrows indicate increased and decreased levels or activity, respectively. Green boxes represent beneficial vascular outcomes, whereas orange boxes highlight the intervention (CoQ10) and oxidative stress-related components.Abbreviations: CoQ10, coenzyme Q10; mtROS, mitochondrial reactive oxygen species; oxLDL, oxidized low-density lipoprotein; NO, nitric oxide; NF-κB, nuclear factor kappa B; FMD, flow-mediated dilation.

Overall, the available evidence suggests that CoQ10 supplementation may improve endothelial function, particularly in older adults and individuals at increased cardiovascular risk. However, current evidence is derived primarily from studies evaluating surrogate vascular endpoints rather than major cardiovascular events or cardiovascular mortality. Therefore, larger, adequately powered randomized controlled trials with longer follow-up are required to determine whether these improvements translate into clinically meaningful cardiovascular benefits (Table 4 and Figure 3) [38,41,42,43,44].

## 6. The Role of MitoQ in Combating Age-Related Vascular Dysfunction Through Mitochondrial Antioxidant Activity

Age-related vascular dysfunction is widely recognized as a major contributor to the development of cardiovascular disease (CVD). Excessive production of mitochondrial reactive oxygen species (mtROS) is considered one of the principal mechanisms underlying vascular injury, leading to endothelial dysfunction and increased arterial stiffness. Consequently, MitoQ, a mitochondria-targeted antioxidant, has attracted considerable scientific interest as a potential therapeutic strategy for improving vascular health [45,46].

MitoQ is a dietary supplement composed of ubiquinol, the reduced form of coenzyme Q10, covalently linked to a triphenylphosphonium (TPP^+^) cation. This structural configuration facilitates its selective accumulation within mitochondria, enabling MitoQ to localize at the primary site of mtROS production and thereby reduce mitochondrial oxidative stress at its source [47].

Preclinical studies have demonstrated that MitoQ supplementation improves endothelial function and reduces arterial stiffness in aged animal models. These promising findings have subsequently been translated into human clinical studies. In a double-blind, randomized, placebo-controlled, crossover trial, Rossman et al. (2018) evaluated the safety and efficacy of MitoQ supplementation in 20 healthy older adults (60–79 years) with impaired endothelial function [47]. Participants received 20 mg/day of MitoQ or placebo for 6 weeks. MitoQ supplementation significantly improved flow-mediated dilation (FMD), a validated surrogate marker of endothelial function, by 42%. Improvements in aortic stiffness, assessed by carotid–femoral pulse wave velocity (cfPWV), were also observed among participants with elevated baseline arterial stiffness. Furthermore, circulating oxidized low-density lipoprotein (ox-LDL) concentrations, a biomarker of oxidative stress, were significantly reduced, whereas no significant changes were observed in endothelium-independent vasodilation or systemic inflammatory markers. These findings suggest that MitoQ may improve vascular function primarily by reducing mitochondrial oxidative stress rather than by exerting broad systemic anti-inflammatory effects [47].

Building on these findings, a phase IIa clinical trial (NCT04851288) is currently evaluating the long-term vascular effects of MitoQ supplementation in a larger cohort of older adults. This randomized, double-blind, placebo-controlled trial includes 90 healthy participants aged ≥60 years who are randomly assigned to receive either MitoQ (20 mg/day) or placebo for 3 months. The primary endpoint is the change in nitric oxide (NO)-mediated endothelium-dependent dilation (EDD), assessed by brachial artery flow-mediated dilation (FMD). Secondary outcomes include measures of arterial stiffness (carotid–femoral PWV, carotid compliance, and β-stiffness index), carotid intima-media thickness, and biomarkers of mitochondrial function and oxidative stress in endothelial cells. The study also evaluates circulating biomarkers of oxidative stress, antioxidant status, and inflammation [47].

**Figure 4:**
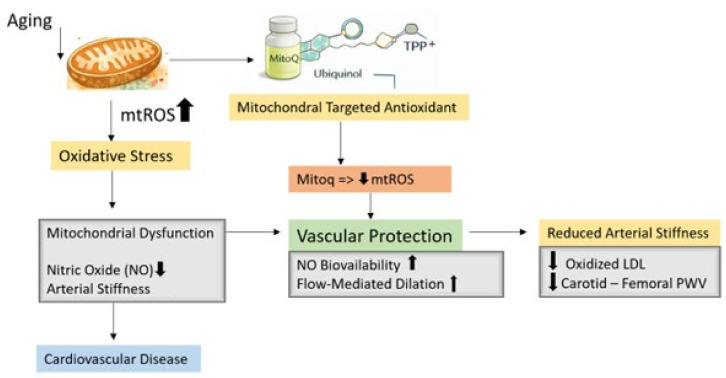
Schematic representation of the role of mitochondrial oxidative stress in age-related vascular dysfunction and the proposed protective mechanisms of MitoQ. Aging is associated with increased mitochondrial reactive oxygen species (mtROS) production, which promotes oxidative stress, mitochondrial dysfunction, reduced nitric oxide (NO) bioavailability, and increased arterial stiffness, ultimately contributing to vascular dysfunction and cardiovascular disease. MitoQ, a mitochondria-targeted antioxidant composed of ubiquinol linked to a triphenylphosphonium (TPP+) cation, selectively accumulates within mitochondria and reduces mtROS production. By lowering mitochondrial oxidative stress, MitoQ is thought to improve NO bioavailability, enhance endothelium-dependent vasodilation, as reflected by increased flow-mediated dilation (FMD), and support vascular function. These effects have been associated with reduced arterial stiffness, lower levels of oxidized low-density lipoprotein (oxLDL), and decreased carotid–femoral pulse wave velocity (PWV). Black arrows indicate the direction of the proposed biological pathway. Upward (↑) and downward (↓) arrows indicate increased and decreased levels or activity, respectively. Green boxes represent beneficial vascular effects, whereas orange boxes represent the intervention (MitoQ) and oxidative stress-related components. The schematic illustration of the MitoQ compound included in the figure was created with the assistance of ChatGPT-5 (OpenAI, https://chatgpt.com; accessed on 20 May 2025). Abbreviations: mtROS, mitochondrial reactive oxygen species; NO, nitric oxide; FMD, flow-mediated dilation; oxLDL, oxidized low-density lipoprotein; PWV, pulse wave velocity; TPP+, triphenylphosphonium cation.

Overall, the available evidence suggests that MitoQ supplementation may improve endothelial function and reduce arterial stiffness through its antioxidant and mitochondrial bioenergetic effects. However, current evidence is derived primarily from randomized controlled trials evaluating surrogate vascular endpoints rather than major cardiovascular events or cardiovascular mortality. Larger, well-designed randomized controlled trials with longer follow-up are required to confirm these findings, further elucidate the underlying mechanisms, and determine whether these improvements translate into clinically meaningful cardiovascular benefits, particularly in older adults at increased cardiovascular risk (Table 5 and Figure 4).

## 7. The Role of Grape Extract Supplementation in Vascular Health

Grape-derived polyphenol extracts have attracted considerable interest as nutraceuticals with potential vascular protective properties. These extracts, particularly those derived from the Aglianico grape variety, are rich in bioactive polyphenols, including resveratrol, catechins, procyanidins, and other phenolic compounds known to exert antioxidant and vasoprotective effects. The clinical study discussed in this review evaluated a commercially available microencapsulated grape polyphenol formulation (Taurisolo®; MB-Med Company, Turin, Italy) which is encapsulated in acid-resistant capsules to protect its bioactive constituents from gastrointestinal degradation and enhance intestinal absorption. Pharmacokinetic studies have shown that grape polyphenol extracts reach peak plasma concentrations approximately 60 min after oral administration, indicating rapid systemic bioavailability [48].

Experimental and clinical studies suggest that grape polyphenols improve endothelial function primarily by enhancing nitric oxide (NO) bioavailability, reducing oxidative stress, and attenuating inflammatory signaling pathways. In addition, favorable effects on lipid oxidation and vascular homeostasis have been reported, supporting the hypothesis that grape-derived polyphenols may contribute to cardiovascular protection beyond their direct antioxidant activity. Although different formulations, including standardized grape polyphenol extracts and microencapsulated preparations, have been evaluated, the available evidence consistently demonstrates improvements in surrogate vascular endpoints, particularly flow-mediated dilation (FMD), whereas evidence demonstrating reductions in major cardiovascular events or cardiovascular mortality remains limited [49,50,51].

One of the proposed mechanisms underlying these vascular benefits involves the reduction in circulating trimethylamine-N-oxide (TMAO) concentrations. TMAO, a metabolite generated by the gut microbiota, has emerged as an independent cardiovascular risk factor associated with endothelial dysfunction and the progression of atherosclerosis through promotion of vascular inflammation and plaque formation [52].

To further investigate these mechanisms, a randomized, double-blind, placebo-controlled clinical trial evaluated the effects of the microencapsulated grape polyphenol formulation in healthy volunteers. Participants completed a 12-week protocol consisting of a 2-week run-in period, an 8-week intervention phase with 400 mg of the formulation administered twice daily or placebo (maltodextrin 400 mg twice daily), followed by a 2-week follow-up period. Endothelial function was assessed by brachial artery flow-mediated dilation (FMD) at baseline, 1 h after acute administration of 800 mg, and after 8 weeks of supplementation [53,54,55]. The formulation significantly improved FMD both acutely (*p* = 0.021) and following long-term supplementation (*p* = 0.019), whereas no significant changes were observed in the placebo group. Furthermore, biomarkers of oxidative stress, including oxidized low-density lipoprotein (ox-LDL) and derivatives of reactive oxygen metabolites (D-ROMs), were significantly reduced by 36.36% (*p* = 0.043) and 23.33% (*p* = 0.008), respectively, suggesting attenuation of oxidative vascular damage [53,54,55].

**Figure 5:**
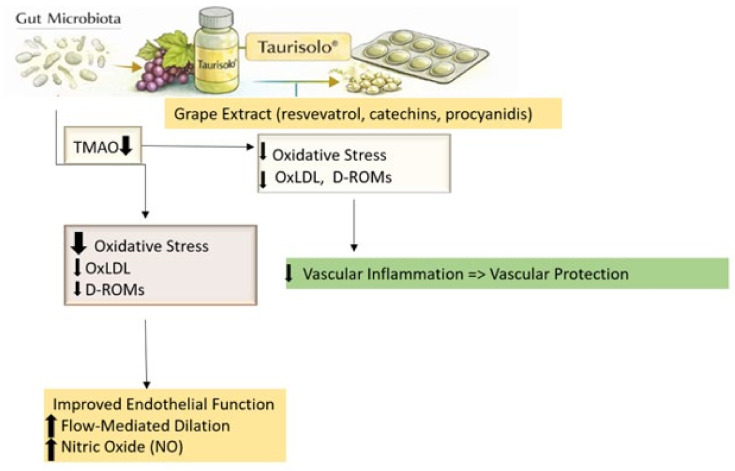
Schematic representation of the vascular protective effects of grape polyphenol extracts, as exemplified by the standardized formulation Taurisolo®, through modulation of gut microbiota-derived metabolites and reduction in oxidative stress. Polyphenolic compounds present in grape extracts, including resveratrol, catechins, and procyanidins, have been associated with reduced circulating levels of trimethylamine N-oxide (TMAO), a gut microbiota-derived metabolite linked to cardiovascular risk. This reduction may contribute to lower oxidative stress, as reflected by decreased levels of oxidized low-density lipoprotein (oxLDL) and derivatives of reactive oxygen metabolites (D-ROMs), as well as attenuation of vascular inflammation. Collectively, these mechanisms may improve endothelial function, enhance nitric oxide (NO) bioavailability, and promote endothelium-dependent vasodilation, reflected by improved flow-mediated dilation (FMD). Black arrows indicate the direction of the proposed biological pathway and interactions. Upward (↑) and downward (↓) arrows indicate increased and decreased levels or activity, respectively. Green boxes represent beneficial vascular effects, whereas orange boxes represent pathological processes or cardiovascular risk factors. The schematic illustration of the standardized grape polyphenol formulation and grape extract included in the figure was created with the assistance of ChatGPT-5 (OpenAI, https://chatgpt.com; accessed on 20 May 2025). Abbreviations: TMAO, trimethylamine N-oxide; oxLDL, oxidized low-density lipoprotein; D-ROMs, derivatives of reactive oxygen metabolites; NO, nitric oxide; FMD, flow-mediated dilation.

Overall, the available evidence suggests that grape-derived polyphenol extracts may improve endothelial function and reduce oxidative stress, primarily through enhancement of NO bioavailability, antioxidant activity, and modulation of gut microbiota-derived metabolites. However, the available clinical evidence is limited and is derived predominantly from studies evaluating a specific microencapsulated grape polyphenol formulation. Furthermore, current evidence is based primarily on surrogate vascular endpoints rather than major cardiovascular events or cardiovascular mortality. Therefore, additional independent, well-designed randomized controlled trials using standardized grape polyphenol formulations are required to confirm the reproducibility, generalizability, and clinical relevance of these findings (Figure 5 and Table 6).

## 8. The Role of Glycocalyx Dietary Supplements in Restoring Endothelial Glycocalyx Integrity and Reducing Arterial Stiffness in Chronic Inflammatory Disease

A key factor in vascular dysfunction, particularly in chronic inflammatory diseases, is the degradation of the endothelial glycocalyx. This is also the main reason why nutritional approaches aimed at preserving or restoring the endothelial glycocalyx are gaining increasing attention because of their therapeutic potential. Among these nutritional interventions is a specialized endothelial glycocalyx-supporting dietary supplement (Endocalyx-Pro^®^, Microvascular Health Solutions). It contains a proprietary blend of bioactive compounds, including fucoidan (a sulfated polysaccharide derived from *Laminaria japonica*), hyaluronic acid, polyphenolic extracts rich in superoxide dismutase (SOD), glucosamine sulfate, and supportive excipients. This mixture has been designed to provide structural substrates, antioxidants, and enzymatic cofactors that synergistically support glycocalyx regeneration and vascular health [56,57,58,59,60,61].

The rationale for endothelial glycocalyx-targeted nutritional interventions is further supported by preclinical evidence demonstrating that supplementation with glycocalyx precursors, including fucoidan, glucosamine sulfate, hyaluronic acid, and antioxidant compounds, promotes glycocalyx restoration and improves vascular function. In experimental models of type 2 diabetes, dietary supplementation with these glycocalyx precursors restored endothelial glycocalyx thickness, improved flow-mediated dilation, and reduced arterial stiffness, while cultured endothelial cells exhibited enhanced glycocalyx regeneration following treatment. Furthermore, low-molecular-weight fucoidan has been shown to protect the endothelial glycocalyx by limiting glycocalyx shedding through modulation of neuraminidase-2 (NEU2), a recently identified regulator of glycocalyx homeostasis, thereby preserving endothelial integrity and reducing vascular injury under diabetic conditions. Nevertheless, the translation of these encouraging experimental findings into clinical practice remains challenging, as a pilot randomized placebo-controlled trial in patients with type 2 diabetes did not demonstrate significant improvements in endothelial glycocalyx integrity or vascular function despite the favorable preclinical results [62,63].

The effectiveness of this endothelial glycocalyx-supporting nutritional formulation has been evaluated in a recent randomized, double-blind, placebo-controlled clinical trial involving patients with psoriasis, a chronic inflammatory skin disorder associated with increased cardiovascular risk [64]. Fifty participants receiving biological therapy were randomly assigned to receive either the nutritional formulation or placebo for 4 months. The primary endpoints included assessments of endothelial glycocalyx thickness through measurements of the perfused boundary region (PBR) and arterial stiffness evaluated through pulse wave velocity (PWV) and the augmentation index (AIx). Secondary outcomes included psoriasis severity measured by the Psoriasis Area and Severity Index (PASI) [65].

The results revealed that although both groups showed comparable improvements in PASI, the intervention group demonstrated significant improvements in vascular markers. Specifically, significant reductions in PBR across multiple vessel size ranges (4–25 μm, 4–9 μm, 10–19 μm, and 20–25 μm) were observed only in the intervention group, indicating increased glycocalyx thickness and integrity. In addition, PWV and AIx were significantly reduced in the intervention group, suggesting improvements in arterial elasticity. Importantly, the correlations between glycocalyx restoration (reduction in PBR) and arterial stiffness parameters (PWV and AIx) were statistically significant, supporting a mechanistic link between glycocalyx integrity and vascular function [65].

The observed vascular benefits are likely attributed to the specific components of the formulation. In particular, fucoidan mimics heparan sulfate, a key structural component of the glycocalyx, and inhibits heparanase, an enzyme responsible for glycocalyx degradation. Hyaluronic acid and glucosamine sulfate contribute to the synthesis of glycosaminoglycans, which are essential for glycocalyx structure. Finally, the antioxidant activity of SOD and polyphenols may protect the endothelium from oxidative stress, a major factor contributing to glycocalyx damage in inflammatory conditions [61,65].

**Figure 6:**
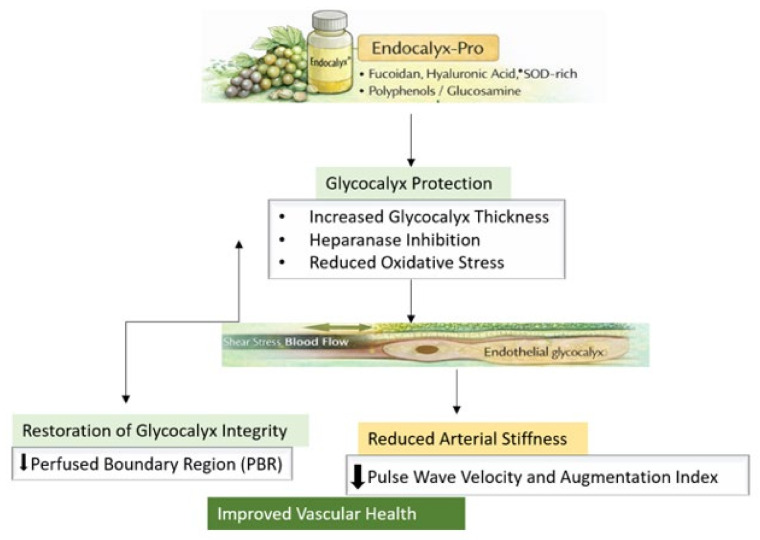
Schematic illustration of the protective effects of the glycocalyx-targeted supplement Endocalyx-Pro on endothelial glycocalyx integrity and vascular function. The supplement contains bioactive components including fucoidan, hyaluronic acid, SOD-rich polyphenols, and glucosamine, which support glycocalyx protection by increasing glycocalyx thickness, inhibiting heparanase activity, and reducing oxidative stress. These mechanisms contribute to restoration of endothelial glycocalyx integrity, reflected by a reduction in the perfused boundary region (PBR). Improved glycocalyx structure is associated with reduced arterial stiffness, as indicated by decreased pulse wave velocity (PWV) and augmentation index (AIx), ultimately promoting improved vascular health. Black arrows indicate the direction of the proposed biological pathway and interactions. Upward (↑) and downward (↓) arrows indicate increased and decreased levels or activity, respectively. Green boxes represent beneficial vascular effects, whereas yellow boxes represent intermediate processes or favorable physiological changes. The schematic illustration of the Endocalyx-Pro supplement and endothelial glycocalyx included in the figure was created with the assistance of ChatGPT-5 (OpenAI, https://chatgpt.com; accessed on 20 May 2025). Abbreviations: PBR, perfused boundary region; PWV, pulse wave velocity; AIx, augmentation index; SOD, superoxide dismutase.

In summary, this study suggests that four months of endothelial glycocalyx-supporting nutritional supplementation may improve glycocalyx integrity and reduce arterial stiffness in patients with psoriasis, without additional changes in clinical inflammation scores. However, the available clinical evidence is based primarily on surrogate vascular endpoints rather than major cardiovascular events or cardiovascular mortality and should therefore be interpreted with caution. Although preclinical evidence consistently supports glycocalyx restoration as a promising therapeutic strategy, clinical findings remain limited and heterogeneous. While improvements in glycocalyx integrity and arterial stiffness have been reported in patients with chronic inflammatory disease, these findings have not yet been consistently reproduced in other patient populations. Therefore, larger independent randomized controlled trials evaluating different endothelial glycocalyx-targeted nutritional interventions are needed to establish their reproducibility, generalizability, and long-term clinical relevance (Table 7 and Figure 6).

## 9. Cardiometabolic Effects of Olive-Derived Bioactive Compounds

### 9.1. Preclinical Evaluation

Preclinical studies have demonstrated that chronic supplementation of mice with olive-derived bioactive compounds at nutritional doses confers significant cardiometabolic benefits. Oleuropein (OL), oleocanthal (OC), and oleanolic acid (OA) markedly reduced myocardial infarct size in both healthy and metabolic syndrome (MS)-burdened mice, whereas hydroxytyrosol (HT) alone did not significantly reduce myocardial infarct size. In the MS model, OL improved glucose homeostasis by lowering fasting glucose and increasing plasma insulin levels, while OA normalized total cholesterol. However, none of the tested compounds affected obesity, triglycerides, or blood pressure.

Three combinatorial treatments—Combo 1 (OL + HT + OA), Combo 2 (OL + HT + OC), and Combo 3 (OL + HT + OC + OA)—demonstrated robust infarct-sparing effects, with Combo 2 additionally improving fasting glucose and insulin levels. Cardiac function remained unchanged, and NMR analysis confirmed the chemical stability of the mixtures. Mechanistic analyses demonstrated that Combo 2 and its constituents reduced apoptosis (indicated by a lower Bax/Bcl-xL ratio) and enhanced antioxidant defenses via upregulation of MnSOD, CAT, and the Nrf2 pathway, without affecting RISK pathway activation or NADPH oxidase (NOX) expression. These findings suggest that cardioprotection is primarily mediated through anti-apoptotic and antioxidant mechanisms rather than suppression of ROS generation via NOX enzymes.

Furthermore, HT, OC, and Combo 2 reduced systemic oxidative stress markers (MDA, ox-LDL, and 3-NT), inhibited NETosis in vitro, and limited atherosclerotic plaque formation and ROS accumulation in ApoE^−^/^−^ mice, demonstrating potent anti-atherogenic activity independent of lipid lowering. Toxicity assessment confirmed the safety of Combo 2 even at threefold higher doses, with no biochemical or histological signs of organ damage [66].

### 9.2. Clinical Evaluation

#### 9.2.1. OL–HT–OC Supplementation in Patients with Chronic Coronary Artery Syndrome

To translate these preclinical findings into clinical practice, a prospective, randomized, double-blind, placebo-controlled, crossover clinical trial, “OL–HT–OC Supplementation in Patients with Chronic Coronary Artery Syndrome,” was conducted in 15 patients with chronic coronary artery syndrome (CCAS). Participants underwent baseline echocardiographic assessment and blood sampling and received either the OL–HT–OC-enriched supplement (Combo 2 equivalent) or a placebo for one month (four capsules daily). After the first month, treatments were crossed over.

Primary endpoints included endothelial glycocalyx thickness, endothelial function, arterial stiffness, coronary function, and left ventricular function. The supplement dosage was based on prior in vivo studies (OL:HT:OC = 20.6:5.9:11.6 mg/kg body weight). The intervention improved vascular, endothelial, and cardiac function, as reflected by improvements in PWV, FMD, CFR, and LVGLS, together with reductions in oxidative stress biomarkers (PCSK9 and ox-LDL), thereby confirming the preclinical findings.

#### 9.2.2. Hydroxytyrosol-Enriched Olive Oil Supplement (OOHT) in Patients with Chronic Coronary Artery Syndrome

The second clinical trial, “Hydroxytyrosol-Enriched Olive Oil Supplement (OOHT) in Patients with Chronic Coronary Artery Syndrome,” evaluated the effect of an olive oil supplement enriched predominantly with hydroxytyrosol (HT) in 30 patients with CCAS. Participants received either four OOHT capsules daily (412.5 mg olive oil + 2.5 mg HT per capsule) or placebo for one month, followed by crossover.

Parameters assessed included the perfused boundary region (PBR) of sublingual microvessels, flow-mediated dilation (FMD), coronary flow reserve (CFR), left ventricular diastolic function assessed by Doppler echocardiography, pulse wave velocity (PWV), and biomarkers of oxidative stress, inflammation, and lipid metabolism. Compared with placebo, hydroxytyrosol-enriched olive oil significantly improved PBR, FMD, CFR, and PWV, while reducing malondialdehyde (MDA), oxidized low-density lipoprotein (ox-LDL), triglycerides, proprotein convertase subtilisin/kexin type 9 (PCSK9), and C-reactive protein (CRP) levels (*p* < 0.05). These vascular improvements may be related to reductions in oxidative stress and inflammation, which may enhance nitric oxide (NO) bioavailability and endothelial function [67,68,69,70].

**Figure 7:**
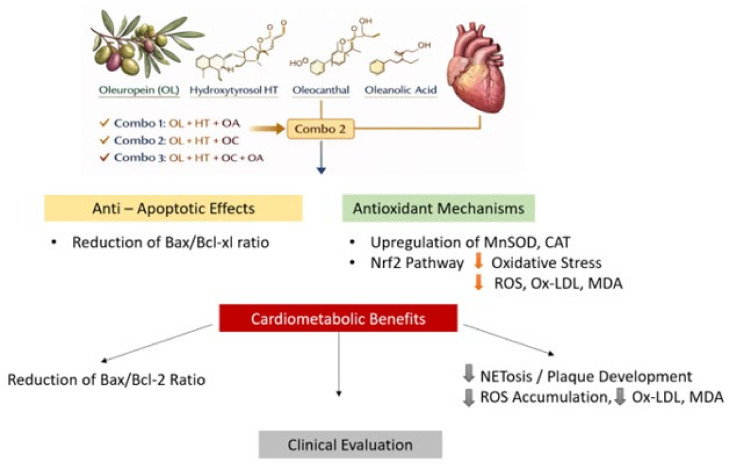
Schematic representation of the cardiometabolic protective effects of olive-derived bioactive compounds. Bioactive molecules present in olives, including oleuropein (OL), hydroxytyrosol (HT), oleocanthal (OC), and oleanolic acid (OA), exert beneficial effects through antioxidant and anti-apoptotic mechanisms. These compounds activate antioxidant signaling pathways such as nuclear factor erythroid 2-related factor 2 (Nrf2), leading to upregulation of antioxidant enzymes including manganese superoxide dismutase (MnSOD) and catalase (CAT). This results in reduced oxidative stress and decreased levels of reactive oxygen species (ROS), oxidized low-density lipoprotein (oxLDL), and malondialdehyde (MDA). Additionally, olive-derived bioactive compounds exert anti-apoptotic effects by reducing the Bax/Bcl-xL ratio and limiting apoptosis. These mechanisms contribute to reduced NETosis, decreased atherosclerotic plaque formation, and overall cardiometabolic protection. Black arrows indicate the direction of the proposed biological pathway and interactions. Upward (↑) and downward (↓) arrows indicate increased and decreased levels or activity, respectively. Green boxes represent beneficial biological effects, whereas yellow boxes represent intermediate mechanisms or signaling pathways, and the red box highlights the overall cardiometabolic benefits resulting from these protective mechanisms. The schematic illustrations of the olive-derived compounds and heart included in the figure were created with the assistance of ChatGPT-5 (OpenAI, https://chatgpt.com; accessed on 20 May 2025). Abbreviations: OL, oleuropein; HT, hydroxytyrosol; OC, oleocanthal; OA, oleanolic acid; ROS, reactive oxygen species; oxLDL, oxidized low-density lipoprotein; MDA, malondialdehyde; MnSOD, manganese superoxide dismutase; CAT, catalase; Nrf2, nuclear factor erythroid 2-related factor 2.

Collectively, the available preclinical and clinical evidence suggests that olive-derived bioactive compounds, particularly the OL–HT–OC combination and hydroxytyrosol-enriched olive oil, may improve endothelial function and vascular surrogate markers through antioxidant and anti-inflammatory mechanisms. However, current clinical evidence is derived primarily from relatively small randomized controlled trials evaluating surrogate vascular endpoints rather than major cardiovascular events or cardiovascular mortality. Therefore, larger, adequately powered randomized controlled trials with longer follow-up are required to determine whether these vascular improvements translate into clinically meaningful cardiovascular benefits (Table 8 and Figure 7).

## 10. S-Allyl Cysteine and a Black Garlic Extract on Vascular Health

It is a reasonable observation that endothelial dysfunction (ED), which in turn reflects impaired regulation of vascular tone and reduced bioavailability of nitric oxide (NO), represents a central factor in hypertension and cardiovascular risk. Among other mediators, hydrogen sulfide (H_2_S) has emerged as an important gasotransmitter with vasodilatory and antioxidant effects, closely interconnected with the NO signaling pathway. Therefore, natural H_2_S donors, which are involved in the pathogenesis of ED, have attracted increasing scientific interest.

S-allylcysteine (SAC), a sulfur-containing amino acid enriched in aged/black garlic, is considered an H_2_S-generating compound with antioxidant and cardiometabolic benefits. In an in vitro model using bovine aortic endothelial cells (BAE-1), SAC was evaluated for its effects on key endothelial health endpoints, including intracellular reactive oxygen species (ROS), H_2_S release, eNOS phosphorylation, and NO production (assessed through fluorescence imaging and Western blot analysis). In parallel, a black garlic extract (BGE) was chemically characterized for SAC and related sulfur-containing amino acids using HPLC-MRM and tested for endothelial H_2_S release [71].

**Figure 8:**
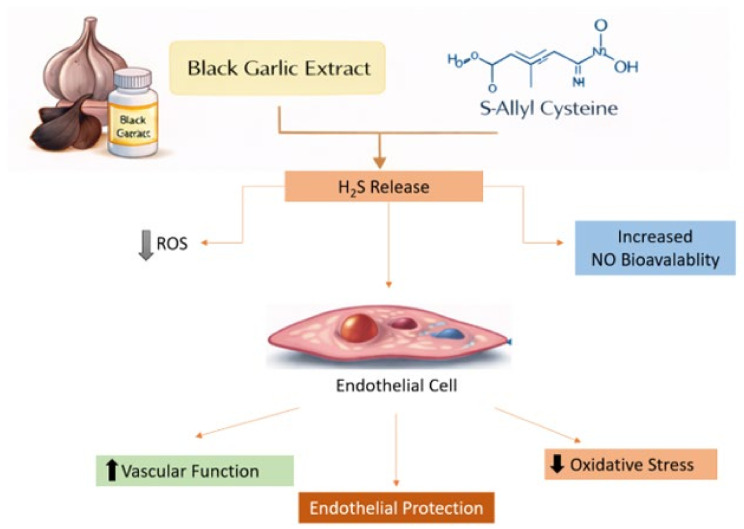
Schematic representation of the mechanisms by which black garlic extract and its major bioactive compound, S-allyl cysteine (SAC), promote vascular health. SAC acts as a hydrogen sulfide (H_2_S) donor, increasing intracellular H_2_S levels in endothelial cells. H_2_S signaling contributes to the reduction in reactive oxygen species (ROS) and enhances nitric oxide (NO) bioavailability through activation of endothelial nitric oxide synthase (eNOS). These effects lead to decreased oxidative stress, improved endothelial function, and enhanced vascular function, ultimately contributing to endothelial protection. Black arrows indicate the direction of the proposed biological pathway and interactions. Upward (↑) and downward (↓) arrows indicate increased and decreased levels or activity, respectively. Green boxes represent beneficial vascular effects, whereas orange boxes represent intermediate mechanisms or physiological processes, and blue boxes indicate increased bioavailability or signaling responses. The schematic illustrations of the black garlic extract, S-allyl cysteine structure, and endothelial cell included in the figure were created with the assistance of ChatGPT-5 (OpenAI, https://chatgpt.com; accessed on 20 May 2025). Abbreviations: H_2_S, hydrogen sulfide; ROS, reactive oxygen species; NO, nitric oxide; eNOS, endothelial nitric oxide synthase; SAC, S-allyl cysteine.

Overall, SAC did not affect cell viability and acted as an effective intracellular H_2_S donor, increasing H_2_S levels comparable to those observed with NaHS. Functionally, SAC reduced menadione-induced ROS, enhanced eNOS phosphorylation, and increased NO release. The chemically characterized BGE also significantly increased intracellular H_2_S levels in BAE-1 cells and did not affect cell viability under short-term exposure conditions. Overall, the available in vitro evidence suggests that S-allyl cysteine (SAC)-enriched, food-derived extracts may influence mechanisms involved in endothelial function, including hydrogen sulfide (H_2_S) signaling, nitric oxide (NO) bioavailability, and oxidative stress. However, these findings are derived exclusively from preclinical experimental models and cannot be directly extrapolated to clinical vascular outcomes. Well-designed human studies are required to determine whether these mechanistic effects translate into meaningful improvements in endothelial function or cardiovascular health (Figure 8 and Table 9).

## 11. The Role of Curcumin in Modulating Vascular Function and Oxidative Stress

Curcumin, a natural polyphenolic compound derived from *Curcuma longa*, has attracted substantial scientific interest due to its potent antioxidant and anti-inflammatory properties and its potential role in modulating vascular function across diverse clinical contexts. Oxidative stress and chronic inflammation are central mechanisms underlying vascular dysfunction, aging, cardiovascular disease (CVD), and chronic kidney disease (CKD). Consequently, curcumin has been investigated as a nutraceutical intervention aimed at improving endothelial function, reducing arterial stiffness, and attenuating inflammatory responses.

Redox homeostasis plays a fundamental role in maintaining vascular integrity and cellular function. Dysregulation of reactive oxygen species (ROS) production and antioxidant defenses contributes to endothelial dysfunction, vascular remodeling, and the development of cardiometabolic disorders. Antioxidants such as polyphenols, including curcumin, have been shown to modulate oxidative pathways by scavenging free radicals, inhibiting NADPH oxidase activity, and activating cytoprotective signaling pathways such as nuclear factor erythroid 2-related factor 2 (Nrf2). These mechanisms are associated with improved nitric oxide (NO) bioavailability, reduced inflammation, and enhanced endothelial-dependent vasodilation, all of which are critical for vascular health [50].

Evidence from experimental and clinical studies suggests that curcumin may exert beneficial effects on vascular function in specific populations. A systematic review investigating menopausal women reported improvements in endothelial function, arterial compliance, and hemodynamic parameters following curcumin supplementation. Clinical trials included in this review demonstrated increases in flow-mediated dilation (FMD) and reductions in systolic blood pressure in postmenopausal women, particularly when curcumin supplementation was combined with regular endurance exercise. Preclinical studies in ovariectomized animal models further supported these findings, showing reduced oxidative stress, improved endothelial integrity, and decreased formation of atherosclerotic lesions. These beneficial effects were attributed primarily to curcumin’s ability to enhance NO bioavailability, suppress inflammatory cytokines such as interleukin-6 (IL-6) and tumor necrosis factor-alpha (TNF-α), and inhibit ROS-generating enzymes.

Despite these promising findings, the vascular benefits of curcumin appear to be context-dependent. A randomized, double-blind, placebo-controlled trial involving patients with stage 3b–4 CKD evaluated the effects of 12-month curcumin supplementation (2000 mg/day) on vascular and cognitive function. The study found no significant improvements in endothelial function, arterial stiffness, or cognitive performance compared with placebo. Specifically, there were no differences in FMD, nitroglycerin-mediated dilation, carotid–femoral pulse wave velocity, or cognitive domain scores after long-term supplementation. However, curcumin significantly reduced circulating IL-6 levels, indicating an anti-inflammatory effect despite the absence of measurable vascular or cognitive improvements. These findings suggest that while curcumin may exert systemic anti-inflammatory actions in CKD, these effects may not translate into clinically meaningful improvements in vascular function in populations with advanced comorbidity and high cardiovascular risk.

The discrepancies between studies may be explained by differences in population characteristics, disease severity, supplementation duration, and curcumin bioavailability. Beneficial vascular effects are more consistently observed in relatively healthy populations or early disease states, such as menopausal women without significant comorbidities, whereas individuals with advanced CKD may exhibit structural vascular changes that are less responsive to antioxidant interventions. Additionally, curcumin’s low oral bioavailability and variable formulation across studies may influence its physiological efficacy.

**Figure 9:**
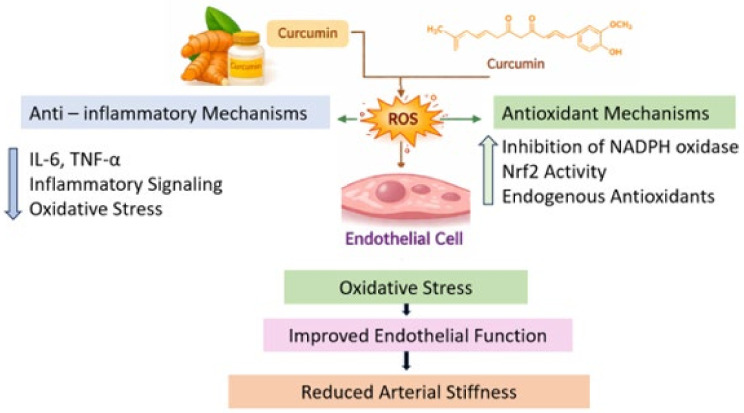
Schematic representation of the mechanisms through which curcumin improves vascular function. Curcumin exerts vascular protective effects through both antioxidant and anti-inflammatory pathways. It reduces reactive oxygen species (ROS) production by inhibiting NADPH oxidase and activating the nuclear factor erythroid 2-related factor 2 (Nrf2) pathway, leading to increased endogenous antioxidant defenses. Additionally, curcumin suppresses inflammatory signaling by reducing pro-inflammatory cytokines such as interleukin-6 (IL-6) and tumor necrosis factor-alpha (TNF-α). These combined mechanisms decrease oxidative stress, improve endothelial function, and contribute to reduced arterial stiffness and improved vascular health. Black arrows indicate the direction of the proposed biological pathway and interactions. Upward (↑) and downward (↓) arrows indicate increased and decreased levels or activity, respectively. Green boxes represent antioxidant and beneficial vascular mechanisms, gray boxes represent anti-inflammatory mechanisms, and orange or pink boxes represent vascular outcomes and physiological effects. The schematic illustrations of curcumin, its chemical structure, and the endothelial cell included in the figure were created with the assistance of ChatGPT-5 (OpenAI, https://chatgpt.com; accessed on 20 May 2025). Abbreviations: ROS, reactive oxygen species; IL-6, interleukin-6; TNF-α, tumor necrosis factor alpha; Nrf2, nuclear factor erythroid 2-related factor 2.

Collectively, current evidence indicates that curcumin possesses significant antioxidant and anti-inflammatory properties with potential vascular benefits under certain physiological and clinical conditions. While curcumin supplementation may improve endothelial function and vascular parameters in populations characterized by moderate oxidative stress and inflammation, its effectiveness in advanced chronic diseases such as CKD remains uncertain. Further large-scale, long-term randomized controlled trials employing standardized curcumin formulations and robust vascular endpoints are required to clarify its therapeutic role in vascular health and disease prevention. [72,73,74] (Table 10 and Figure 9).

## 12. Overall Evidence Summary

To facilitate comparison across the nutritional supplements reviewed, Table 11 provides an overall summary of the current level of evidence, the principal vascular effects reported, and the major limitations of the available studies. This overview highlights the variability in the strength and consistency of the evidence, emphasizing that while several supplements demonstrate beneficial effects on surrogate markers of vascular function, the overall quality of the available evidence remains heterogeneous and is frequently limited by small sample sizes, short intervention periods, product-specific formulations, and the absence of hard cardiovascular outcomes. Accordingly, although current evidence suggests that several nutritional supplements may improve surrogate markers of vascular function, adequately powered, well-designed randomized controlled trials using standardized interventions and clinically meaningful cardiovascular outcomes are required to establish their long-term clinical value.

## 13. Safety Considerations and Clinical Applicability

Although the nutraceuticals discussed in this review have generally demonstrated favorable safety profiles in clinical studies, several practical considerations should be taken into account before their routine clinical use. Most randomized controlled trials have reported only mild adverse effects, predominantly gastrointestinal symptoms, while serious adverse events have been uncommon. Nevertheless, the duration of supplementation in most available studies has been relatively short, limiting conclusions regarding long-term safety.

The effective dose ranges varied considerably among supplements. L-citrulline has typically been administered at doses ranging from 2 to 10 g/day, cocoa flavanols at approximately 450–900 mg/day, CoQ10 between 100 and 300 mg/day (although doses up to 1200 mg/day have been reported as well tolerated), whereas doses and formulations of grape polyphenols, olive polyphenols, and glycocalyx-targeting supplements differed substantially between studies. Therefore, direct comparisons among studies should be interpreted cautiously.

Potential interactions with cardiovascular medications should also be considered. Several nutraceuticals, including CoQ10, L-citrulline, cocoa flavanols, and olive polyphenols, may influence blood pressure or endothelial function and could theoretically enhance the effects of antihypertensive therapies. Likewise, polyphenol-rich supplements may interact with anticoagulant or antiplatelet medications, although clinically significant interactions remain insufficiently documented. Consequently, supplementation should be individualized, particularly in older adults and patients with multiple comorbidities or polypharmacy.

Another important consideration is the variability in bioavailability and product standardization. The biological activity of many nutraceuticals depends on the manufacturing process, formulation, dose, and individual differences in absorption and metabolism. Furthermore, beneficial effects observed with standardized dietary supplements should not necessarily be extrapolated to the consumption of the corresponding whole foods, which differ substantially in composition and bioactive compound concentrations.

Overall, nutritional supplementation should be regarded as an adjunct rather than a replacement for guideline-directed lifestyle modification and pharmacological therapy. Healthcare professionals should consider the available clinical evidence, patient characteristics, concomitant medications, and product quality before recommending these interventions in routine clinical practice.

## 14. Conclusions

Overall, selected nutraceuticals appear to modestly improve markers of vascular function, particularly endothelium-dependent dilation (FMD), arterial stiffness (PWV), and, in some studies, blood pressure. These effects are mainly attributed to mechanisms involving increased nitric oxide bioavailability, reduction in oxidative stress, improved mitochondrial redox balance, and preservation of the endothelial glycocalyx.

However, the strength of the available evidence varies considerably among different supplements and formulations, as most studies are small in scale, short in duration, and primarily focused on intermediate surrogate vascular endpoints rather than major cardiovascular outcomes. In clinical practice, nutritional supplementation should be regarded as an adjunct to lifestyle modification and guideline-directed cardiovascular prevention strategies, with careful consideration of patient selection, potential interactions, and the regulatory framework governing health claims.

Future research should incorporate standardized vascular phenotyping, adequately powered randomized controlled trials, and transparent reporting of product composition, dosage, and adherence to confirm the potential benefits of nutritional supplements on vascular function. In addition, combining supplements with complementary mechanisms of action, including anti-inflammatory compounds and agents that support endothelial structure and glycocalyx integrity, represents a promising research direction that warrants further investigation.

It should be emphasized that most of the available evidence summarized in this review is based on surrogate vascular endpoints, including flow-mediated dilation (FMD), pulse wave velocity (PWV), blood pressure, endothelial glycocalyx integrity, and biomarkers of oxidative stress. Although improvements in these parameters are associated with better vascular health, current evidence remains insufficient to conclude that the reviewed nutritional supplements reduce major adverse cardiovascular events or cardiovascular mortality. Therefore, larger, well-designed randomized controlled trials with longer follow-up and hard clinical endpoints are required before definitive clinical recommendations can be made.

## Figures and Tables

**Table 1:** Summary of randomized controlled trials evaluating watermelon (L-citrulline) supplementation and vascular function.

Study (Ref)	Study Design	Population (*n*)	Intervention	Comparator	Dose	Duration	Primary Vascular Endpoint	Main Findings	Limitations
Meta-analysis of 10 RCTs [5,7]	Meta-analysis of randomized controlled trials	10 RCTs (*n* = 564); adults aged 22–58 years (healthy or prehypertensive/obese)	Watermelon (L-citrulline-rich) supplementation	Control/placebo	2.7–6 g/day L-citrulline (juice: 3.4 g/day; acute studies: 0.8–6.9 g)	16 days–6 weeks (acute studies: 45 min–8 h)	PWV, AIx, MAP, PWA, postprandial blood flow	↓ PWV (−0.9 m/s, *p* < 0.001); no significant changes in AIx, MAP, or PWA; ↓ postprandial glucose (−0.6 mmol/L, *p* < 0.001); ↑ plasma L-arginine and L-citrulline	Small individual trials; heterogeneous interventions and doses; surrogate vascular endpoints; limited long-term follow-up

**Table 2:** Clinical studies investigating the effects of L-citrulline supplementation on endothelial function and vascular health.

Study (Ref)	Study Design	Population (*n*)	Intervention	Comparator	Dose	Duration	Primary Vascular Endpoint	Main Findings	Limitations
Maharaj et al. (2022) [8]	Randomized, placebo-controlled trial	25 hypertensive postmenopausal women	L-citrulline supplementation	Placebo	10 g/day	4 weeks	FMD, cfPWV, aortic BP	↑ L-arginine; ↑ FMD (Δ1.4 ± 2.0% vs. −0.5 ± 1.7%; *p* = 0.03); ↓ aortic diastolic BP and MAP; no significant change in cfPWV (*p* = 0.07)	Small sample size; short intervention; surrogate vascular endpoints
Jaime et al. (2022) [10]	Randomized, placebo-controlled trial	16 older adults	L-citrulline supplementation	Placebo	6 g/day	14 days	SBP, pulse pressure, augmented pressure, and wave reflection	↓ Brachial and aortic SBP, pulse pressure, augmented pressure, and wave reflection during the cold pressor test	Small sample size; short intervention; acute physiological stress model
Shatanawi et al. (2020) [11]	Randomized controlled trial	25 patients with type 2 diabetes mellitus	L-citrulline supplementation	Control	2 g/day	1 month	Plasma NO levels, arginase activity	↓ Arginase activity (21%); ↑ plasma NO levels (38%); no significant change in HbA1c	Small sample size; surrogate vascular endpoints; limited follow-up
Barkhidarian et al. (2019) [12]	Systematic review and meta-analysis	Mixed populations	L-citrulline supplementation	Not applicable	3–9 g/day	1–17 weeks	Blood pressure	↓ Systolic BP (~4 mmHg); dose-dependent reduction in diastolic BP (≥6 g/day); substantial heterogeneity among studies	Considerable heterogeneity in study populations, doses, and intervention duration

**Table 3:** Randomized controlled trials evaluating cocoa flavanol supplementation and vascular function.

Study (Ref)	Study Design	Population (*n*)	Intervention	Comparator	Dose	Duration	Primary Vascular Endpoint	Main Findings	Limitations
West et al. (2014) [34]	Randomized controlled trial	30 middle-aged overweight or obese adults (mean age: 51 years)	Dark chocolate plus sugar-free cocoa beverage	Control beverage	814 mg flavanols/day	6 weeks	Brachial artery diameter, hyperemic blood flow, AIx	↑ Brachial artery diameter before hyperemia and at peak dilation; ↑ resting and peak hyperemic blood flow; ↓ AIx in women	Small sample size; sex-specific effects
Heiss et al. (2015) [35]	Randomized, controlled, double-masked trial	42 healthy men (22 young and 20 older adults)	Cocoa flavanol beverage	Control beverage	900 mg flavanols/day (146 mg monomers)	4 weeks	FMD, PWV, total peripheral resistance, AIx	↑ FMD; ↓ PWV; ↓ total peripheral resistance; ↓ aortic AIx; ↑ vasodilatory capacity in older adults	Relatively small sample size; healthy participants; surrogate vascular endpoints
Rassaf et al. (2016) [36]	Randomized, double-blind, placebo-controlled trial	49 patients with end-stage renal disease receiving hemodialysis	Cocoa flavanol-rich beverage	Placebo	900 mg flavanols/day	4 weeks	FMD, diastolic blood pressure	↑ FMD; ↓ diastolic blood pressure; ↑ heart rate compared with placebo	Small sample size; disease-specific population; surrogate vascular endpoints
Sansone et al. (2015) [37]	Randomized, controlled, double-masked trial	100 healthy men and women (mean age: 47 years)	Cocoa flavanol beverage	Control beverage	900 mg flavanols/day (146 mg monomers)	4 weeks	FMD, SBP, DBP, PWV, AIx	↑ FMD; ↓ office and central systolic and diastolic blood pressure; ↓ PWV; ↓ AIx compared with the control group	Healthy population; surrogate vascular endpoints

**Table 4:** Clinical evidence on coenzyme Q10 supplementation and endothelial function.

Study (Ref)	Study Design	Population (*n*)	Intervention	Comparator	Dose	Duration	Primary Vascular Endpoint	Main Findings	Limitations
Daei et al. (2024) [41]	Systematic review and meta-analysis of 12 randomized controlled trials	489 participants (12 RCTs)	CoQ10 supplementation	Placebo or control	100–300 mg/day	4–24 weeks	FMD, ICAM-1, VCAM-1	↑ FMD; no significant changes in ICAM-1 or VCAM-1; greater improvement among participants aged >55 years, those with BMI ≥ 26 kg/m^2^, and those receiving supplementation for ≥8 weeks	Heterogeneity in study populations, CoQ10 doses, and intervention durations; relatively small individual trials; surrogate vascular endpoints

**Table 5:** Clinical studies evaluating the effects of MitoQ supplementation on vascular function.

Study (Ref)	Study Design	Population (*n*)	Intervention	Comparator	Dose	Duration	Primary Vascular Endpoint	Main Findings	Limitations
Rossman et al. (2018) [47]	Randomized, double-blind, placebo-controlled, crossover trial	20 healthy older adults (60–79 years) with impaired endothelial function (FMD < 6%)	MitoQ supplementation	Placebo	20 mg/day	6 weeks	FMD, cfPWV, ox-LDL	↑ Plasma MitoQ concentrations; ↑ FMD by 42% compared with placebo (*p* < 0.05); ↓ aortic stiffness in participants with elevated baseline cfPWV; ↓ ox-LDL; no significant changes in endothelium-independent vasodilation or inflammatory markers	Small sample size; healthy older adults; surrogate vascular endpoints
Murray et al. (Ongoing; NCT04851288) [47]	Phase IIa randomized, double-blind, placebo-controlled trial	90 healthy adults aged ≥60 years	MitoQ supplementation	Placebo	20 mg/day	3 months	FMD, cfPWV, carotid compliance, β-stiffness index	Ongoing trial; results not yet available. Primary endpoint: FMD. Secondary endpoints: mitochondrial ROS, arterial stiffness, NO bioavailability, oxidative stress, and mitochondrial function	Ongoing study; no published efficacy results available

**Table 6:** Clinical study evaluating grape polyphenol extract supplementation and vascular function.

Study (Ref)	Study Design	Population (*n*)	Intervention	Comparator	Dose	Duration	Primary Vascular Endpoint	Main Findings	Limitations
Stein et al. (1999) [50]	Prospective clinical intervention study	15 patients with coronary artery disease	Purple grape juice	Baseline (within-subject comparison)	7.7 mL/kg/day	14 days	FMD, LDL oxidation	↑ FMD (2.2% to 6.4%; *p* = 0.003); ↓ LDL susceptibility to oxidation (+34.5%), supporting antioxidant and endothelial protective effects	Small sample size; no placebo group; short intervention; surrogate vascular endpoints
Lekakis et al. (2005) [51]	Randomized, placebo-controlled trial	30 men with coronary heart disease	Red grape polyphenol extract	Placebo	600 mg (single dose)	Acute (120 min)	FMD	↑ Brachial artery FMD, peaking at 60 min (4.52% vs. 2.64% with placebo; *p* < 0.001), indicating rapid improvement in endothelial function	Acute intervention; surrogate vascular endpoint; relatively small sample size
Li et al. (2013) [49]	Meta-analysis of nine controlled trials	Healthy adults and individuals with cardiovascular risk factors	Grape polyphenol supplementation	Control or placebo	Single doses ranging from ~150–1400 mg total polyphenols	Acute (30–120 min)	FMD	↑ Acute FMD during the first 2 h after supplementation; greater responses in participants with cardiovascular risk factors, with peak effects at 60 min in patients with coronary artery disease and smokers	Heterogeneity in polyphenol source, dose, and study design; acute outcomes only
Martelli et al. (2021) [55]	Randomized, placebo-controlled clinical trial	Healthy volunteers (*n* = 50)	Microencapsulated grape polyphenol formulation	Placebo	400 mg twice daily	8 weeks	FMD, RHI, ox-LDL, D-ROMs	↑ Acute and chronic FMD; ↓ ox-LDL (36.4%; *p* = 0.043); ↓ D-ROMs (23.3%; *p* = 0.008); no significant changes in RHI, indicating improved endothelial function and reduced oxidative stress	Single proprietary formulation; healthy population; relatively small sample size; surrogate vascular endpoints; independent replication required

**Table 7:** Clinical study evaluating endothelial glycocalyx-supporting nutritional supplementation in patients with psoriasis.

Study (Ref)	Study Design	Population (*n*)	Intervention	Comparator	Dose	Duration	Primary Vascular Endpoint	Main Findings	Limitations
Ikonomidis et al. (2024) [65]	Randomized, double-blind, placebo-controlled trial	50 patients with psoriasis receiving biological therapy	Endothelial glycocalyx-supporting nutritional formulation	Placebo	2556 mg/day (4 capsules/day)	4 months	PBR, PWV, AIx	↓ PBR; ↓ PWV; ↓ AIx, indicating improved endothelial glycocalyx integrity and reduced arterial stiffness; no significant between-group difference in PASI score	Single proprietary formulation; disease-specific population; moderate sample size; surrogate vascular endpoints
Smith et al. (2024) [63]	Randomized, double-blind, placebo-controlled pilot trial	22 veterans with type 2 diabetes mellitus	Dietary supplementation with glycocalyx precursors (fucoidan, glucosamine sulfate, hyaluronic acid, and SOD/polyphenols)	Placebo	3712.5 mg/day	8 weeks	PBR, FMD, cfPWV	No significant improvements in PBR, brachial or femoral FMD, cfPWV, plasma nitrite levels, or other vascular function indices compared with placebo	Pilot study; small sample size; short intervention; disease-specific population; surrogate vascular endpoints

**Table 8:** Preclinical and clinical studies investigating olive-derived bioactive compounds and vascular function.

Study (Ref)	Study Design	Population (*n*)	Intervention	Comparator	Dose	Duration	Primary Vascular Endpoint(s)	Main Findings	Limitations
Christodoulou et al. (2024) [66]	Prospective, randomized, double-blind, placebo-controlled, crossover trial	15 patients with chronic coronary artery syndrome (CCAS)	Olive-derived bioactive compound combination (OL–HT–OC; Combo 2 equivalent)	Placebo	Four capsules/day (OL:HT:OC = 20.6:5.9:11.6 mg/kg body weight)	1 month (crossover)	Endothelial glycocalyx thickness, FMD, PWV, CFR, LVGLS	↑ FMD, CFR, and LVGLS; ↓ PWV; ↓ PCSK9 and ox-LDL compared with placebo	Small sample size; crossover design; surrogate vascular endpoints
Ikonomidis et al. (2023) [70]	Randomized, placebo-controlled, crossover trial	30 patients with chronic coronary artery syndrome (CCAS)	Hydroxytyrosol-enriched olive oil	Placebo	Four capsules/day (412.5 mg olive oil + 2.5 mg hydroxytyrosol per capsule)	1 month (crossover)	PBR, FMD, CFR, PWV	↓ PBR and PWV; ↑ FMD and CFR; ↓ MDA, ox-LDL, triglycerides, PCSK9, and CRP; no significant change in blood pressure	Small sample size; crossover design; surrogate vascular endpoints

**Table 9:** Preclinical studies evaluating the effects of S-allyl cysteine and black garlic extracts on endothelial function.

Study (Ref)	Study Design	Experimental Model	Intervention	Primary Outcome	Main Findings	Limitations
Geddo et al. (2023) [71]	In vitro experimental study with chemical profiling	BAE-1 endothelial cells; HPLC-MRM analysis	S-allyl cysteine (SAC; 100 μM) and SAC-enriched black garlic extract (100 μM SAC equivalent)	Cell viability, H_2_S release, ROS production, eNOS phosphorylation, NO release, sulfur compound profiling	No reduction in cell viability; ↑ H_2_S release; ↓ ROS under oxidative stress; ↑ eNOS phosphorylation; ↑ NO release; quantified SAC and related sulfur compounds; no short-term cytotoxicity	In vitro model; formulation-specific findings; no in vivo or clinical validation; findings require confirmation in animal models and randomized clinical trials

**Table 10:** Preclinical and clinical evidence on curcumin supplementation and vascular function.

Study (Ref)	Study Design	Population (*n*)	Intervention	Comparator	Dose	Duration	Primary Vascular Endpoint	Main Findings	Limitations
Mad Azli et al. (2024) [74]	Systematic review of seven studies	Menopausal women and ovariectomized animal models	Curcumin supplementation	Not applicable	150–1000 mg/day (humans); 50–100 mg/kg/day (animals)	8–16 weeks (humans); 4–12 weeks (animals)	FMD, blood pressure, arterial stiffness	↑ FMD; ↓ systolic blood pressure; improved vascular structure; greater effects when combined with exercise; anti-inflammatory effects were consistently reported	Considerable heterogeneity; inclusion of both human and animal studies; variable doses and intervention durations
Gimblet et al. (2024) [72]	Randomized, double-blind, placebo-controlled trial	88 adults with stage 3b–4 chronic kidney disease	Curcumin supplementation	Placebo	2000 mg/day	12 months	FMD, arterial stiffness	No significant improvement in FMD or arterial stiffness; ↓ IL-6	Disease-specific population (CKD); surrogate vascular endpoints unchanged despite anti-inflammatory effects

**Table 11:** Overall summary of the evidence supporting the effects of nutritional supplements on vascular function.

Supplement	Highest Level of Evidence	Main Vascular Effects	Overall Quality of Evidence	Main Limitations
L-citrulline	Meta-analysis + RCTs	↑ FMD, ↓ BP	Moderate	Small RCTs; surrogate endpoints
Cocoa flavanols	Multiple RCTs	↑ FMD, ↓ PWV, ↓ BP	Moderate–High	Mainly surrogate outcomes
Coenzyme Q10	Meta-analysis of RCTs	↑ FMD	Moderate	Heterogeneity; no effect on ICAM-1/VCAM-1
MitoQ	Small RCT + ongoing trial	↑ FMD	Low–Moderate	Limited clinical evidence
Grape polyphenols	Small RCT	↑ FMD, ↓ oxLDL	Low	Commercial formulation; small sample
Olive-derived bioactive compounds	Small crossover RCTs	↑ FMD, ↓ PWV	Low	Small studies; surrogate endpoints
Curcumin	Systematic review + RCT	Mixed findings	Low–Moderate	Heterogeneous populations and interventions
Endothelial glycocalyx-supporting formulations	Small RCT	Improved glycocalyx integrity; ↓ PWV	Low	Product-specific evidence
S-allyl cysteine/Black garlic	Preclinical studies	↑ NO, ↓ ROS	Preclinical	Lack of human clinical studies

## Data Availability

No new data were created or analyzed in this study.
